# Fatty Infiltration of Mitral Valve: A Rare Case Report and Review of Literature

**DOI:** 10.7759/cureus.6144

**Published:** 2019-11-13

**Authors:** Geetika Goyal, Syed H Abbas, Michael A Diamond, Rajiv Pulinthanathu

**Affiliations:** 1 Pathology, Saint Barnabas Medical Center, Robert Wood Johnson Barnabas Health, Livingston, USA; 2 Pathology, Monmouth Medical Center, Long Branch, USA

**Keywords:** fatty infiltration, mitral valve

## Abstract

Adipose tissue is a normal anatomical finding in the heart but fat infiltration in cardiac valves is extremely rare with very few cases reported in the literature. We report a case of fatty infiltration in a mitral valve replaced for mitral valve prolapse causing severe mitral regurgitation in a 65-year-old male who presented with shortness of breath and upper respiratory tract infection. The histopathological examination of the mitral valve revealed sheets of mature adipocytes in the spongiosa layer leading to replacement of collagen and elastic fibers. The presence of adipocytes in the prolapsed valve could be considered to arise from the proliferation of pluripotent valvular interstitial cells. Herein, we have reviewed the previously reported cases of fatty infiltration in the mitral valve and discussed the pathogenesis and effect on valvular function.

## Introduction

Fatty tissue is a normal component of the heart and is usually located in the epicardium, pericardium, and interatrial septum, but not a normal anatomic finding in the heart valves. The various pathological conditions associated with fatty deposition in the heart include lipomatous metaplasia in myocardium scar tissue secondary to ischemic heart disease, fat in the myocardium of right ventricle in arrhythmogenic right ventricular dysplasia, and in obese patients with metabolic syndrome [1-2]. Adipose tissue infiltration in cardiac valves is exceptionally rare. We report a case of fatty infiltration in an incompetent mitral valve in a 65-year-old male who underwent mitral valve replacement for severe mitral valvular regurgitation. We have discussed the pathogenesis of fat infiltration in mitral valves and have reviewed the previously reported cases of fat infiltration in the mitral valve. 

## Case presentation

A 65-year-old male was admitted to the emergency department with acute shortness of breath and chest discomfort. The symptoms were gradually progressive over the past few days. He had an episode of acute upper respiratory infection in the past week and was on decongestants for symptoms of cold and congestion. The patient tested negative for influenza A and B. He had a significant medical history of mitral valve prolapse, benign prostatic hyperplasia, and hernia repair. He had no significant history of smoking, alcohol or drug abuse. Upon examination, the patient’s vitals were within normal limits. The patient was not overweight and had a bodyweight of 72 kg. The jugular venous pressure was normal. The heart rate was regular and in normal sinus rhythm. A soft ejection systolic heart murmur was present. The breath sounds were present and were equal on both sides of the chest.

The lab results were significant for brain natriuretic peptide (BNP) levels elevated up to 407 pg/ml (normal up to 100 pg/ml), normocytic normochromic anemia, leukocytosis with increased neutrophils, and mild thrombocytopenia consistent with bacterial pneumonia. However, blood cultures were negative, likely due to empiric antibiotic treatment. The BNP levels returned to normal after the valve replacement.

**Figure 1:**
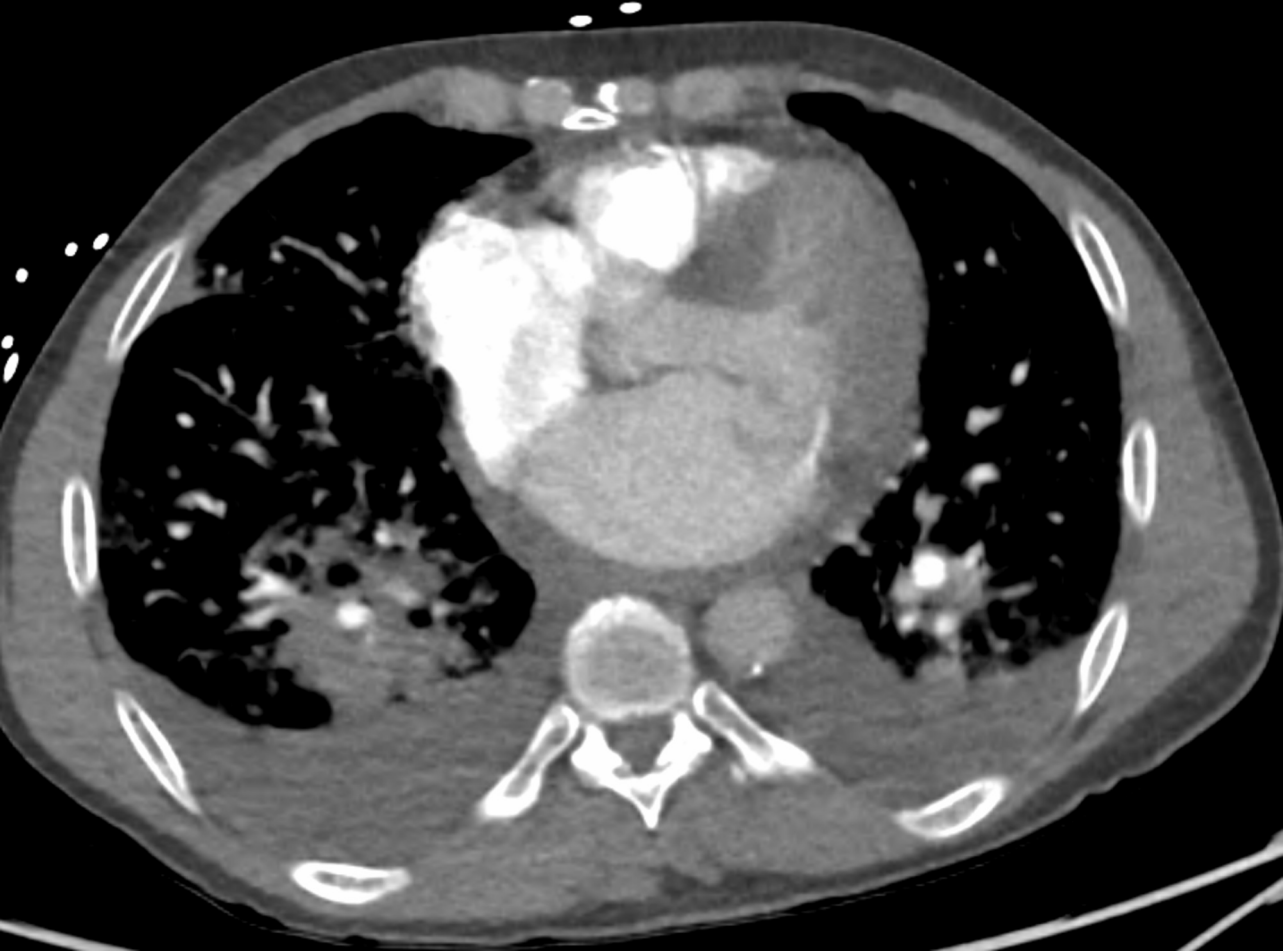
Computerized tomographic angiogram of lungs showing bilateral opacities and consolidation in both the lungs and bilateral pleural effusions

The electrocardiogram (ECG) showed normal sinus rhythm with premature atrial contractions, prolonged QT interval, and ST depression followed by subsequent elevation in the lateral leads. No arrhythmias were present. The chest X-ray showed bilateral interstitial infiltrates. The computed tomographic angiogram (CTA) of the chest showed diffuse bilateral airspace opacities, consistent with multifocal pneumonia (Figure 1). Small bilateral pleural effusions were present.

No aortic aneurysm was seen. Pulmonary embolism was ruled out. There was borderline pulmonary artery enlargement reflecting a degree of pulmonary artery hypertension. A potential right renal parapelvic cyst was identified. The patient was admitted for treatment and further testing. The treatment for bilateral pneumonia was started with oxygen through high flow nasal cannula and antibiotics including ceftriaxone and azithromycin. The patient was started on heparin to provide prophylaxis for deep vein thrombosis.

In light of his previous significant history of mitral valve prolapse, a transthoracic echocardiogram (TTE) was performed that showed a normal-sized left ventricle with concentric mild left ventricular hypertrophy as well as mild right ventricular and moderate right atrial dilatation. The left ventricular ejection fraction was found to be normal (0.6-0.65). He had grade III diastolic dysfunction with a restrictive pattern, consistent with markedly increased left atrial pressure. No left atrial mass or thrombus was visualized. There was mild mitral valve thickening with a flail posterior mitral leaflet. There were no vegetations on the mitral valve. No rupture of chordae tendineae was noted. The mitral regurgitant jet was anteriorly directed consistent with posterior leaflet pathology. There was severe mitral valve regurgitation as well as mild tricuspid and aortic valve regurgitation. There was no pericardial effusion. However, severe pulmonary hypertension was noted; the coronary angiography revealed normal coronary arteries with right coronary artery dominant circulation in the heart. The aortic root was normal in size.

The patient’s medications during hospitalization included aspirin, famotidine, furosemide, and acetaminophen. The mitral valve replacement was done. The post-procedure echocardiography confirmed the bioprosthetic valve in place. No valvular or para-valvular regurgitation was seen. The left ventricular function was normal. The patient had normal sinus rhythm post-procedure upon ECG. The patient recovered and was discharged in good condition on postoperative day seven without any complications.

**Figure 2:**
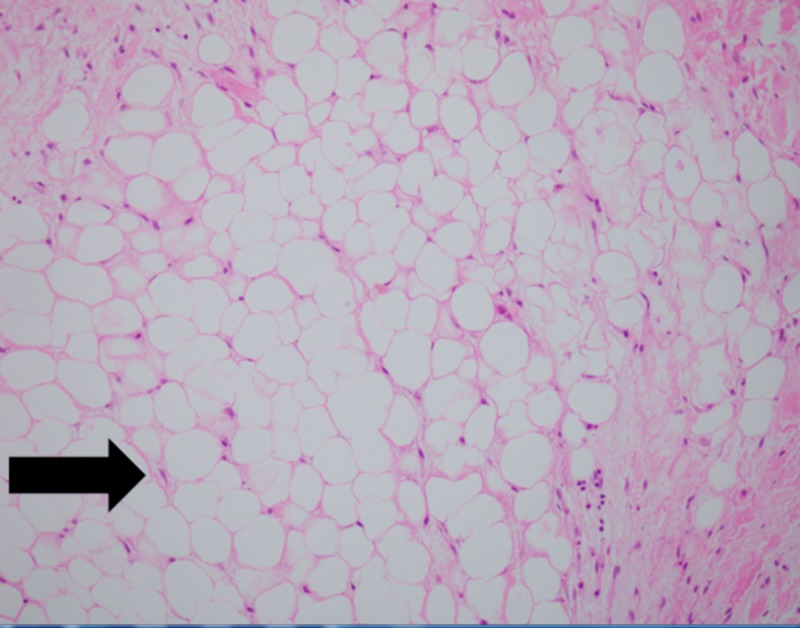
Microscopic image of the mitral valve showing replacement of the spongiosa layer with mature adipocytes (H&E, 40X)

**Figure 3:**
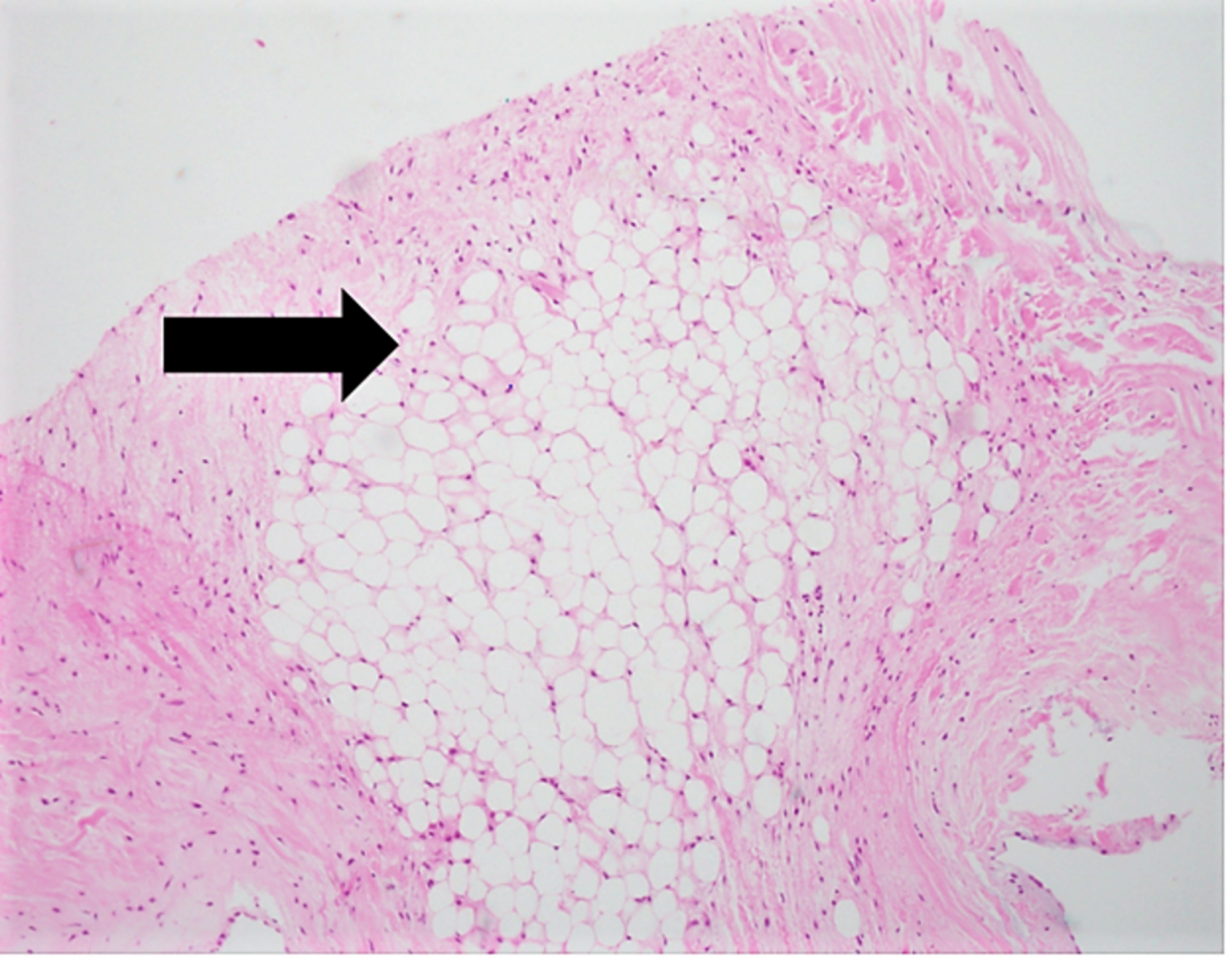
Hematoxylin and eosin-stained micrograph of the mitral valve showing the replacement of spongiosa layer with mature adipocytes (H&E, 20X)

The native mitral valve was received in the pathology department for histopathological examination. The macroscopic examination of the valve showed two valvular cusps measuring 3.0 x 2.0 x 0.1 cm each along with multiple separate pieces measuring 0.3 x 0.2 x 0.2 cm, which included chordae tendineae measuring 2.5 x 2.0 cm. The surface of the cusps showed yellow nodules with focal calcifications. No other gross abnormalities were noted. The microscopic examination revealed aggregates of mature adipocytes in the spongiosa layer of the valve, replacing the collagen and elastic fibers (Figures 2-3).

**Figure 4:**
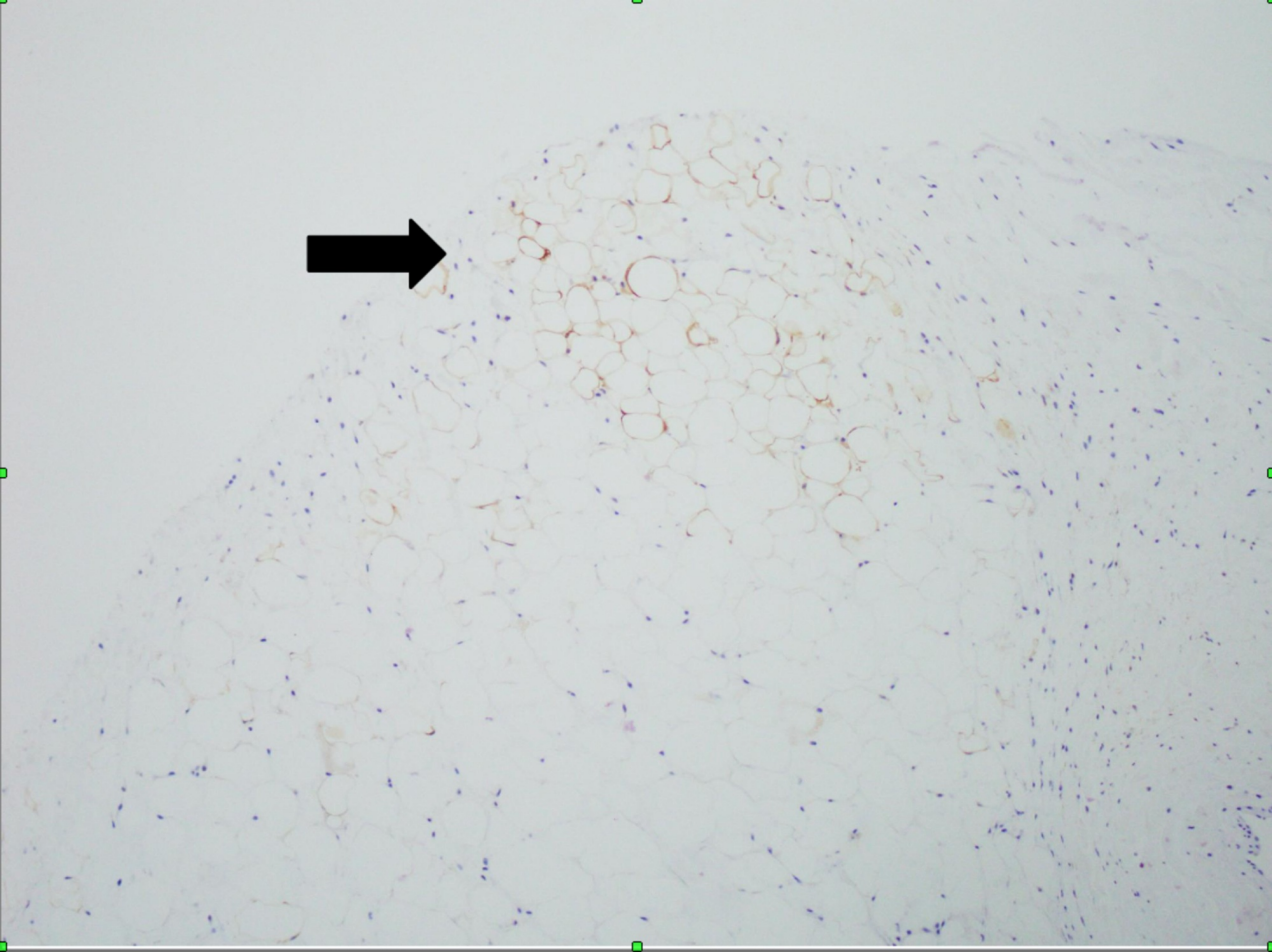
Immunohistochemical staining with S-100 in mitral valve showing the replacement of the spongiosa layer with mature adipocytes (S-100,20X)

There was no extension of adipocytes to the valve annulus. Calcific atherosclerosis was seen, but there were no foamy macrophages or vegetations. The immunohistochemical stain for S-100 was positive and showed adipocytes (Figure 4).

## Discussion

As a normal anatomic component, adipose tissue is seen in various locations in the heart, including the epicardium, pericardium, and interatrial septum. The fat deposition in subendocardium in right ventricles may be seen in pernicious anemias as a characteristic linear pattern in the wall of the right ventricle described as "tigering" [3].

The normal mitral valve apparatus is a dynamic three-dimensional system that allows the inflow of blood from the left atrium to the left ventricle during diastole and unidirectional pumping of blood into the aorta during systole preventing any backflow into the left atrium. The key structural components of the mitral valve are the mitral annulus, the mitral valve leaflets, the chordae tendineae, and papillary muscles attached to the left ventricular wall. The proper functioning of the mitral valve depends upon the harmonious interplay of all mitral valve components. An imbalance in the structure and function of any of these components leads to valvular dysfunction. The normal mitral valve has two leaflets: anterior and posterior.

Histologically, the mitral valve leaflets have a trilaminar structure, which consists of three layers: ventricularis or fibrosa layer, spongiosa layer, and atrialis layer. The ventricularis or fibrosa layer of the leaflets is seen in continuity with the ventricles and is hemodynamically exposed to left ventricular pressures. This layer is composed of dense collagen and imparts mechanical stability to the valve. The spongiosa layer is rich in water-absorbent proteins that protect the edges of the valve leaflets and ensures a tight valvular seal. The atrialis layer contains collagen and elastin proteins vital for the remodeling of leaflets.

The anterior and posterior mitral leaflets differ significantly in the distribution of the layers. The dense collagen layer of ventricularis forms majority of the anterior leaflet thickness and contributes to the strength of the anterior leaflet as compared to the thinner and more flexible posterior mitral leaflet.

The extracellular matrix turnover is very slow in a healthy mitral valve due to dormant interstitial cells and rudimentary vascular and lymphatic system. The activation and proliferation of interstitial cells leading to neovascularization and remodeling of the extracellular matrix may be induced by the physiological or pathologic-induced stress on the leaflets [4].

There have been a few case reports describing fatty infiltration in cardiac valves. As per the literature review, lipid involvement of aortic valves may be seen with aging and fat infiltration of the aortic valves has been seen in structurally normal valves [5]. The fatty infiltration in the mitral valve in most cases has been seen in association with incompetent mitral valvular function confirmed upon echocardiography with associated symptoms of mitral regurgitation. Echocardiography is the preferred imaging modality for the diagnosis and evaluation of valvular structural and functional abnormalities. The imaging may reveal yellow nodules or masses on the lines of the closure of the cusps of the semilunar valve [6]. The fat infiltration in the present case was less prominent and the nodules were not appreciable upon imaging. Focal yellow tiny nodules were noted on the macroscopic examination of the valves.

Upon microscopic examination, the fat distribution was seen in spongiosa layers of both cusps and did not appear to reach the valvular annulus. Similar to the present case, G Meredith et al. reported fat in an incompetent aortic valve with valvular regurgitation and with fat involving and limited to the spongiosa of all three cusps of the valve [7].

In atrioventricular valves, rare cases of fatty infiltration have been described involving the mitral and tricuspid valves. These include an abnormal mass in the left atrium and mitral valve found upon autopsy of a 12-year-old child clinically misinterpreted as an atrial myxoma. The surgical exploration revealed that the mass arose from the posterior mitral valve leaflet and was diagnosed as fibro lipoma upon histopathological examination [8]. A similar case has been reported in a five-year-old child who had echocardiography for a membranous ventricular septal defect; an incidental nodule was identified on the anterior leaflet, clinically misinterpreted as myxoma, and diagnosed as lipoma upon histopathology [9]. The cardiac lipomas have been reported not limited to the cardiac valves and involving the papillary muscles and causing valvular dysfunction [10].

In the present case, the mitral valve was incompetent with the prolapse of the posterior mitral leaflet causing severe mitral regurgitation. Our patient had mitral valve replacement for severe mitral regurgitation. Mitral valve replacement has been done in previously reported cases of mitral valve lipomas [11-12].

The pathogenesis of fat infiltration in cardiac valves has not been fully understood and many etiologies have been proposed. These include developmental and hamartomatous. The developmental theory seems to be less likely as not many cases have been reported in pediatric age groups. The fat infiltration has been linked to the hamartomatous origin and associated with structural abnormalities including prolapsing, cystic or windsock malformations in the valves. Prolapse and malformation of the tricuspid valve could be explained due to chronic exposure to increased right ventricular pressures. In the present case, the hamartomatous origin of fat infiltration may be considered based on the prolapsed nature of valve leaflets [13].

Lipomatous hamartomas have been reported in mitral valves with infiltration into the myocardium [14]. In the present case, the fat was limited to the valves, and no infiltration into adjacent structures was noted. The well-understood theory is the differentiation of pre-existing valvular interstitial progenitor cells as a result of various valvular insults. The valvular injury secondary to ischemia, infections, therapeutic, and diagnostic procedures may lead to the proliferation of valvular interstitial cells. These cells are known to be pluripotent in nature and may differentiate into osteogenic, chondrogenic, myofibrogenic, and adipogenic lineages. These cells comprise extracellular matrix fibroblasts, myofibroblasts, and smooth muscle cells. In cases of injury, the myofibroblasts are the predominant cell type [15]. The fat infiltration in our case could be explained by the proliferation of the valvular interstitial cells.

## Conclusions

This article highlights a potential diagnostic pitfall wherein fatty infiltration can be misdiagnosed as lipoma. Despite the rarity of these lesions, fat infiltration in the mitral valves has been associated with mitral regurgitation and could be explained by the proliferation of valvular interstitial cells secondary to valvular insults. Nevertheless, further research may be instrumental to elucidate the pathogenesis of fatty infiltration in atrioventricular cardiac valves.
